# Engineering hyaluronic acid-based cryogels for CD44-mediated breast tumor reconstruction

**DOI:** 10.1016/j.mtbio.2022.100207

**Published:** 2022-01-24

**Authors:** Mahboobeh Rezaeeyazdi, Thibault Colombani, Loek J. Eggermont, Sidi A. Bencherif

**Affiliations:** a Department of Chemical Engineering, Northeastern University, Boston, MA, 02115, USA; b Department of Bioengineering, Northeastern University, Boston, MA, 02115, USA; c Harvard John A. Paulson School of Engineering and Applied Sciences, Harvard University, Cambridge, MA, 02138, USA

**Keywords:** Tumor models, Breast cancer, Cryogels, Hyaluronic acid, CD44

## Abstract

Breast cancer is a major health concern worldwide and is the leading cause of cancer-related death among American women. Traditional therapies, such as surgery, chemotherapy, and radiotherapy, are usually ineffective. Furthermore, cancer recurrence following targeted therapy often results from acquired drug resistance. Therefore, more realistic tumor models than monolayer cell culture for drug screening and discovery in an *in vitro* setting would facilitate the development of new therapeutic strategies. Toward this goal, we first developed a simple, rapid, low-cost, and high-throughput method for generating uniform multi-cellular tumor spheroids (MCTS) with controllable size. Next, biomimetic cryogel scaffolds fabricated from hyaluronic acid (HA) were utilized as a platform to reconstruct breast tumor microtissues with aspects of the complex tumor microenvironment in three dimensions. Finally, we investigated the interactions between the HA-based cryogels and CD44-positive breast tumor cells, individually or as MCTS. We found that incorporating the adhesive RGD peptide in cryogels led to the formation of a monolayer of tumor cells on the polymer walls, whereas MCTS cultured on RGD-free HA cryogels resulted in the growth of large and dense microtumors, more similar to native tumor masses. As a result, the MCTS-laden HA cryogel system induced a highly aggressive and chemotherapy drug-resistant tumor model. RGD-free HA-based cryogels represent an effective starting point for designing tumor models for preclinical research, therapeutic drug screening, and early cancer diagnosis.

## Graphical abstract

**Figure:**
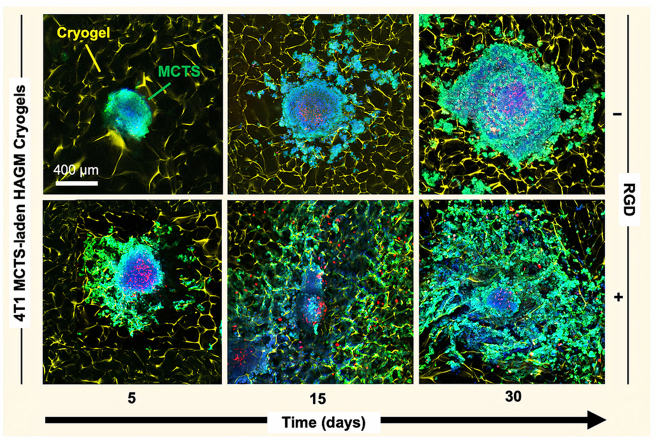

## 1 Introduction

Breast cancer is the most common cancer among American women and the second leading cause of cancer-related death [1]. In 2021, an estimated 281,550 new cases of invasive breast cancer were expected to be diagnosed in the US, and 43,600 breast cancer-related deaths are expected [2]. Despite improvements in treatment methods, there is still a high failure rate mainly due to tumor invasion and metastasis [3]. Furthermore, traditional therapies such as surgery, chemotherapy, and radiotherapy, can be disfiguring and potentially induce local or systemic toxicity. This has spurred the development of breast cancer targeted therapies. However, targeted options for triple-negative breast cancer patients are limited. In addition, cancer resistance or relapse may still occur, and metastatic breast cancer remains predominantly incurable and at the foremost concern for cancer patients [4,5].

The local tumor microenvironment (TME) plays an important role in mediating therapeutic resistance and immune escape, resulting in cancer progression [[6], [7], [8]]. The breast TME is a complex milieu, including structural extracellular matrix (ECM) proteins, signaling molecules, and stromal cells, and provides dynamic signals that influence cell growth, migration, and differentiation [[9], [10], [11]]. Breast cancer invasion is a multi-step process characterized by altered cellular adhesion, cell motility, and invasion through the ECM [12]. Signals transmitted to the tumor cells from the peritumoral stroma can influence their activity, proliferation, and motility [[13], [14], [15]].

Hyaluronic acid (HA) is a major component of breast tumor ECM that has pivotal roles in malignant tumor progression and invasion [[16], [17], [18], [19], [20]]. HA not only provides physical support for tumor cells but also regulates cell-cell adhesion, cell migration, growth, and differentiation [17]. Furthermore, HA can protect tumor cells from immune system attacks by forming pericellular coats [21,22]. Therefore, several tumor cells produce or induce HA biosynthesis by releasing growth factors and cytokines [23]. Tumor cells mainly bind to HA through the cluster of differentiation 44 (CD44), a widely expressed transmembrane protein that is a cell surface marker in breast carcinomas [[24], [25], [26]]. Engagement of CD44 by HA is associated with diverse cellular functions such as cell adhesion, migration, and invasion, which all contribute to cancer progression and metastasis [24].

*In vitro* tumor models that accurately recreate the native TME are needed to effectively screen anti-cancer drugs and develop strategies to overcome therapeutic resistance. Although two-dimensional (2D) cell culture has been a dominant method in many studies, 2D culture has limitations in mimicking the morphology, migration, metabolism, signaling, and gene expression of native tumor cells [27]. Therefore, efforts have been made to develop new *in vitro* tumor models that better mimic the organization of *in vivo* tumors. This has led to the emergence of three-dimensional (3D) tumor models, particularly multi-cellular tumor spheroids (MCTS). Advantages of *in vitro* tumor models in 3D include our ability to study cell-cell and cell-ECM interactions and analyze the influence of the microenvironment on cellular differentiation, proliferation, apoptosis, and gene expression. MCTS possess several characteristics of *in vivo* tumors and their microenvironments, such as cell-cell signaling, ECM production, and hypoxia [28]. Consequently, MCTS can be used to reproduce the 3D architecture of solid tumors, filling the gap between 2D cultured cells and animal models. Furthermore, MCTS mirror avascular tumor nodules and micro-metastases, making them an effective tool for investigating cell function in an avascular TME [27]. In addition, MCTS could be used for drug screening, studying tumor-immune cell interactions, and developing new therapeutic strategies [29,30].

Several strategies have been developed to generate MCTS such as the hanging drop method and the suspension flask technique [12,13]. However, most of these methods are complex and laborious, often resulting in MCTS with low shape and size predictability as well as poor uniformity [31,32]. A simpler alternative consists in producing MCTS using a liquid overlay technique (LOT) in well plates. In this approach, cells are first seeded on agarose-based concave surfaces and then optionally cultured under orbital shaking. Although this approach provides a high throughput method for generating MCTS with more controllable size and shape, they still do not accurately emulate the native tumor ECM [[33], [34], [35], [36], [37], [38], [39]]. Biomaterial scaffolds, such as hydrogels, can help recapitulate key features of the TME, including tumor cells and stromal cells architecture in 3D, ECM composition, and soluble factor gradients [[40], [41], [42]]. Nonetheless, hydrogels often exhibit a mesoporous structure that limits cell motility, invasion, and the diffusion of small biomolecules (e.g., oxygen, nutrients), while the native ECM surrounding cells typically exhibits a hierarchical porous structure [[43], [44], [45], [46], [47], [48], [49], [50], [51], [52], [53]]. Therefore, a macroporous hydrogel system with a highly interconnected network is required to model more accurately the mechanisms of breast cancer invasion and metastasis. Furthermore, current *in vitro* tumor models that involve hydrogels generally rely on integrin-mediated interactions of tumor cells with ECM-derived peptidic ligands such as arginine-glycine-aspartic acid (RGD). Consequently, these scaffolds may not be suitable for studying CD44/HA-mediated signaling pathways in the context of CD44-positive breast cancer development.

**Fig. 1:**
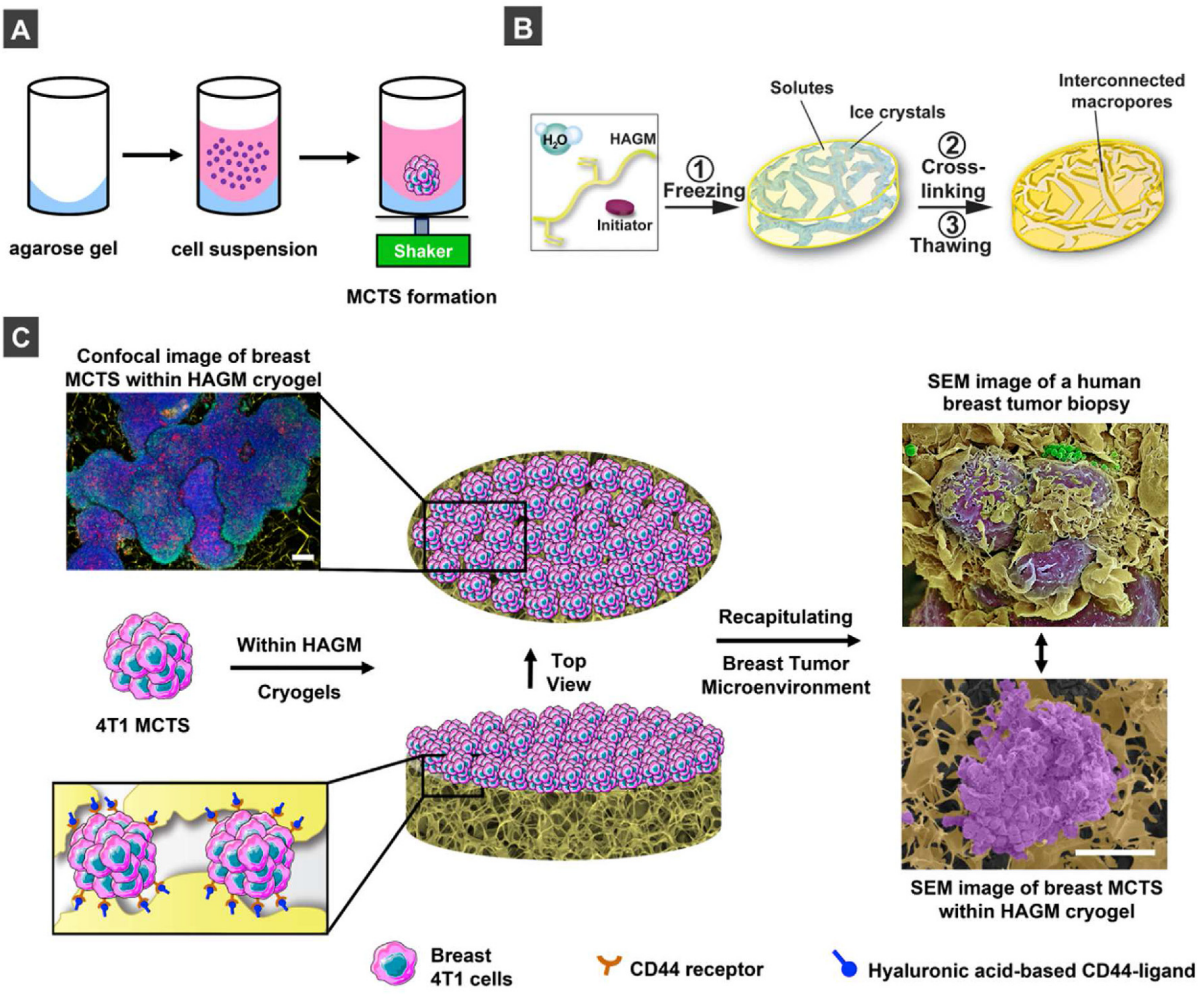
**Illustration describing a model of breast TME with HAGM cryogel.** (A) Schematic depicting 4T1 MCTS formation through orbital shaking of 100 ​cells for 5 days, resulting in spherical MCTS with a diameter of about 400 ​μm. (B) Schematic representation of cryogel fabrication using a cryopolymerization process. An initiator is mixed with an aqueous solution of 4% HAGM, deposited in a mold, and placed at −20 ​°C, leading to phase separation of solutes and ice crystals. HAGM crosslinks around ice crystals, leaving behind an interconnected macroporous network after thawing. (C) Recapitulating a complex breast TME by culturing 4T1 MCTS on HAGM cryogel for up to 30 days. A complex TME is formed through the unique interaction between CD44 receptors of 4T1 cells and HA. The colored SEM image of 4T1 MCTS (purple) within HAGM cryogel (goldish) shows a similar TME when compared to a human breast tumor biopsy (credit: Steve Gschmeissner/Science Photo Library. Reproduced with permission). Scale bars ​= ​200 ​μm. (For interpretation of the references to color in this figure legend, the reader is referred to the Web version of this article).

The aim of this study was to investigate the impact of biochemical cues inherently present in a HA-based macroporous matrix on CD44-positive breast cancer modeling. The preparation of 3D porous scaffolds with a chemical composition that resembles the native ECM could provide a more realistic *in vitro* tumor model for predictive analysis of tumor progression and metastasis, drug efficacy, and new cancer treatment strategies [[54], [55], [56], [57], [58], [59], [60]]. We hypothesized that macroporous HA-based cryogels could provide CD44-positive breast cancer MCTS with robust physical support, allowing simultaneous cell-cell and cell-ECM interactions. Furthermore, these cryogels would help us investigate the role of CD44/HA-mediated signaling in mammary carcinoma progression and metastasis. This study reports on (*i*) a simple, fast, and low-cost method for generating uniform breast cancer MCTS with tunable sizes as depicted in Fig. 1A, (*ii*) a strategy to better mimic CD44-positive breast TME *in vitro* using HA-based cryogels as illustrated in Fig. 1B–C, the (*iii*) consequences of using inappropriate adhesion moieties in biomaterials design to develop cancer models, and *(iv*) the potential of cryogels as a versatile platform to recapitulate key features of aggressive breast tumors.

## 2 Materials and methods

### 2.1 Synthesis of methacrylated HA

To prepare HA amenable to cryogelation, methacrylated HA (HAGM) was synthesized by the reaction of HA with glycidyl methacrylate as previously described [61]. Briefly, at room temperature (RT), 1 ​g of HA (Sigma-Aldrich) was dissolved in 200 ​mL of phosphate-buffered saline (PBS, pH 7.4, Sigma-Aldrich) and subsequently mixed with 67 ​mL of dimethylformamide (Sigma-Aldrich), 13.3 ​g of glycidyl methacrylate (Sigma-Aldrich), and 6.7 ​g of triethylamine (Sigma-Aldrich). After 10 days of reaction, the solution was precipitated in an excess of acetone. The product was then filtered, dried overnight in a vacuum oven at RT, and stored at −20 ​°C until further use.

### 2.2 Synthesis of PEGDM

Polyethylene glycol dimethacrylates (PEGDM) was prepared by reacting 4 ​k polyethylene glycol (PEG, Sigma-Aldrich) and methacrylic anhydride (MA, Sigma-Aldrich). Briefly, at RT, 5 ​g of PEG, 0.34 ​g of MA, and 0.2 ​mL of triethylamine were mixed in 15 ​mL of anhydrous dichloromethane. After 2 days of reaction, the solution was precipitated in an excess of diethyl ether. The product was then filtered, dried overnight in a vacuum oven at RT, and stored at −20 ​°C until further use.

### 2.3 Synthesis of rhodamine-conjugated HAGM

Aminated HAGM was prepared by grafting adipic acid dihydrazide (AAD, Sigma-Aldrich) to HAGM via a carbodiimide-mediated reaction. Briefly, 1 ​g of HAGM was dissolved in 100 ​mL of MES buffer (pH 5.5). A total of 4 ​g of AAD and 90 ​mg of 1-(3-dimethylaminopropyl)-3-ethylcarbodiimide hydrochloride (Sigma-Aldrich) were subsequently added to the mixture while stirring at RT. After 4 ​h of reaction, the modified polymer was precipitated in an excess of acetone, filtered, and dried in a vacuum oven at RT. Next, aminated HAGM was fluorescently labeled with 5/6-carboxy-tetramethyl-rhodamine succinimidyl ester (NHS-Rhodamine, Thermo Fisher). Aminated HAGM (1 ​g) and NHS-Rhodamine (10 ​mg) were dissolved in 100 ​mL of sodium bicarbonate buffer (NaHCO_3_, Sigma-Aldrich, pH 8.5), protected from light using aluminum foil, and then stirred overnight at RT. The product was then precipitated in an excess of acetone, filtered, dried in a vacuum oven overnight at RT, and stored at −20 ​°C until further use.

### 2.4 Synthesis of acrylate-PEG-G_4_RGDSP

Acrylate-PEG-G_4_RGDSP was synthesized by coupling amine-terminated GGGGRGDSP (G_4_RGDSP) peptide (Peptide 2.0) to acrylate-PEG-N-hydroxysuccinimide (JenKem Technology) comonomer (molar ratio 1:1). The reaction proceeded for 4 ​h in sodium bicarbonate buffer (pH 8.5) at RT and the solution was subsequently freeze-dried to obtain acrylate-PEG-RGD. The product was then stored at −20 ​°C until further use.

### 2.5 Fabrication of cryogels

Redox-induced free-radical cryopolymerization was used to fabricate cryogels in deionized water (diH_2_O) at −20 ​°C [61,62]. HAGM was dissolved in diH_2_O to the desired final concentration of 4% (w/v) in the presence of 0.56% (w/v) ammonium persulfate (APS, Sigma-Aldrich) and 0.14% (w/v) tetramethylethylenediamine (TEMED, Sigma-Aldrich). To fabricate RGD-containing cryogels, 0.8% (w/v) acrylate-PEG-RGD was added to the polymer solution. The precooled polymer solution at 4 ​°C was quickly poured into Teflon molds, transferred to a freezer at −20 ​°C, and allowed to cryopolymerize for 15 ​h. Finally, the square-shaped cryogels (4 ​mm ​× ​4 ​mm ​× ​1 ​mm) were thawed at RT, washed rigorously with diH_2_O, and stored at 4 ​°C until further use.

### 2.6 Physical characterization of hydrogels and cryogels

#### 2.6.1 Mechanical properties

Young's moduli were determined using an Instron testing system (Instron 5944, Instron, Norwood, MA, USA). Cylindrical cryogels (6 ​mm diameter, 6 ​mm height) were dynamically deformed (at a constant rate) between two parallel plates for 10 cycles with a strain rate of 10% per minute. Compressive strain (mm) and load (N) were then measured at the 8th cycle using an Instron's Bluehill 3 software (Instron 5944, Instron, Norwood, MA, USA). The Young's modulus was defined as the slope of the stress-strain curve. The gel cylinders were kept hydrated in PBS (pH 7.4) throughout the tests.

#### 2.6.2 Swelling ratio

The swelling ratio was determined using a conventional gravimetric procedure. Cylindrical hydrogels and cryogels were prepared and immersed in PBS for 24 ​h before each measurement. The equilibrium mass swelling ratio (Q_M_) was calculated by dividing the mass of fully swollen cryogel by the mass of freeze-dried cryogel. The cryogels were carefully washed in diH_2_O before freeze-drying to remove the salt content.

#### 2.6.3 Pore connectivity

The pore connectivity was evaluated using a water-wicking technique. Fully hydrated hydrogels and cryogels disks (6 ​mm diameter, 1 ​mm height) were first weighed on an analytical scale. A Kimwipe was then used to wick away free water within the interconnected pores. The partially dehydrated hydrogels and cryogels were weighed again. The recorded values were then used to calculate the degree of pore connectivity as previously reported [62].

### 2.7 Microstructural imaging

Cryogel samples were imaged by scanning electron microscopy **(**SEM) for a qualitative assessment of their macroporous architecture. For sample preparation, cryogels were freeze-dried, adhered onto sample stubs using carbon tape, and coated with platinum in a sputter coater. Samples were then imaged using secondary electron detection on a Hitachi S-4800 scanning electron microscope (Hitachi High-Technology Corporation, Tokyo, Japan) operating at 5 ​kV and 10 ​μA.

### 2.8 Fluorescence imaging

Confocal fluorescence microscopy was used to assess the viability and attachment of cells and MCTS within cryogel scaffolds. Samples were imaged using a Leica TCS SP5 X WLL Confocal Microscope (Buffalo Grove, IL, United States) and analyzed using ImageJ software (Version 1.52e, Bethesda, MD, USA).

### 2.9 Cell viability and attachment

Murine breast cancer cells (NIH/4T1, CRL- 2539, ATCC, Rockville, MD, USA) were cultured in Roswell Park Memorial Institute Medium (RPMI 1640) supplemented with 10% fetal bovine serum (FBS, Sigma-Aldrich), 100 ​μg/mL penicillin (Thermo Fisher), and 100 ​μg/mL streptomycin (Thermo Fisher) and incubated under standard conditions (5% CO_2_, 95% air, 37 ​°C). Human triple-negative breast cancer cells (MDA-MB-231, ATCC) were cultured in Leibovitz's L-15 Medium supplemented with 10% FBS and subsequently incubated under standard conditions (5% CO_2_, 95% air, 37 ​°C). Prior to cell seeding, square-shaped cryogels (4 ​mm ​× ​4 ​mm ​× ​1 ​mm) were sanitized with 70% ethanol for 30 ​min, then washed several times with sterile water. Cryogels were mechanically compressed on a sterile gauze to partially remove free water under sterile conditions before cell seeding. Ten microliters of cell suspension at the desired cell concentration (3 ​× ​10^6^ ​cells/mL) in a complete culture medium were added dropwise on top of each cryogel and then incubated for 4 ​h. Cell-laden cryogels were supplemented with 1 ​mL of fresh complete media for the extent of the experiment and incubated under standard conditions (5% CO_2_, 95% air, 37 ​°C). Cell distribution was noted to be homogeneous throughout the constructs. Cell viability was determined by fixable dead cell staining. After 1, 3, or 5 days of incubation, cells were treated with a far-red fixable dead cell staining according to manufacturer's instructions (ViaQuant™, Genecopoeia). Cells were then fixed with 4% formaldehyde solution (PFA, Sigma-Aldrich) for 30 ​min at RT and washed with PBS. Prior to imaging, cells were permeabilized with 0.1% Triton X-100 in PBS (Sigma-Aldrich) for 5 ​min, then stained with DAPI (Sigma-Aldrich), Alexa Fluor 488 or 647-phalloidin (Cell Signaling Technology), and anti-Zonula Occludens (ZO)-1 Alexa Fluor 488 antibody (clone ZO1-1A12, Invitrogen) according to manufacturers' protocols. For each cryogel, 4 representative sections were imaged by confocal microscopy. Cell viability was determined as the fraction of viable cells over the total number of cells.

### 2.10 MCTS formation

The wells of regular 96-well flat-bottom cell culture plates were covered with a sterile agarose solution (15 ​mg/mL). The agarose was then left in a sterile environment for 30 ​min at RT to solidify. One hundred microliters of a cell suspension in Dulbecco's Modified Eagle Medium (DMEM) supplemented with 10% FBS, 100 ​μg/mL penicillin, and 100 ​μg/mL streptomycin) at various concentrations (from 10^3^ up to 10^4^ ​cells/mL) were then transferred to each agarose-coated well. The cells were incubated under standard conditions (5% CO_2_, 95% air, 37 ​°C) on an orbital shaker (200 ​rpm) to form a single MCTS on each well of 96-well plates. The same method was used to form MCTS under static conditions without orbital shaking.

### 2.11 MCTS-laden cryogels

MCTS (100 ​cells/well, 5-day incubation time) were freshly harvested from each well of a 96-well plate with a precut pipette tip and seeded on top of RGD-free and RGD-containing disc-shaped HAGM cryogels (17 ​mm diameter, 1 ​mm thickness). MCTS-laden cryogels were then cultured in a 12-well plate up to 30 days in DMEM supplemented with 10% FBS, 100 ​μg/mL penicillin, and 100 ​μg/mL streptomycin and incubated under standard conditions (5% CO_2_, 95% air, 37 ​°C).

### 2.12 Cell proliferation

The proliferation of cells cultured in 2D and 3D was assessed by alamarBlue® assay (GeneCopoeia) following the manufacturer's instructions. At various time points (1 or 3 days), cells were first incubated in 600 ​μM alamarBlue® for 1 ​h and then the supernatant was collected and analyzed using a SpectraMax Plus Microplate Reader. Absorbance values at 570 ​nm were recorded to quantify cell proliferation rates.

### 2.13 Flow cytometry analysis

Brilliant violet 605-*anti*-mouse/human CD44 antibody and brilliant violet 605-rat IgG2b isotype antibody were purchased from BioLegend (San Diego, CA). Following three washes with 1 ​× ​PBS containing 1% bovine serum albumin (BSA), 3T3 and 4T1 cells were incubated with antibodies on ice for 30 ​min, washed again, and then characterized by flow cytometry (BD FACSCalibur DxP upgraded, Cytek Bioscience, Fremont, Ca, USA). Flow cytometry data were processed using FlowJo (Tree Star Inc., Ashland, OR) analysis software.

### 2.14 Anti-cancer drug testing

To assess acquired resistance to chemotherapy, a non-lytic assay (alamarBlue®) was performed to monitor cancer cell viability when subjected to doxorubicin (DOX) treatments. Briefly, 4T1 cells cultured in 2D, 4T1-laden RGD-free and RGD-containing HAGM cryogels, MCTS on agarose gel, and MCTS-laden RGD-free and RGD-containing HAGM cryogels were incubated in complete media containing Dox at various concentrations (0–50 ​μM, Tocris Bioscience). The media was changed every 24 ​h for 3 days. Cell viability was monitored daily using alamarBlue®) following the manufacturer's protocol. After 3 days of treatment, the fraction of viable cells for each drug concentration was calculated and normalized to untreated cells. For 2D cultures, anti-cancer drug sensitivity experiments were performed in standard 24-well plates (Corning Costar 3596, Corning Inc.). To assess the diffusion of low molecular weight molecules such as Dox (543.52 ​g/mol) within hydrogels and cryogels, the scaffolds were loaded with 200 ​μL of Trypan blue (872.88 ​g/mol, Sigma-Aldrich) and photographed at various time points (T ​= ​0 ​s, 5 ​s, 15 ​s, 30 ​s, 5 ​min, and 1 ​h).

### 2.15 Gene expression assays

The gene expression of cancer cells was assessed by reverse transcription quantitative polymerase chain reaction PCR (RT-qPCR). The targets were the following: (*i*) hypoxia-inducible factors 1α (HIF1α; Mm00468869_m1), a key regulator of genes involved in the cellular response to hypoxia; (*ii*) apoptosis regulator B-cell lymphoma-2 (BCL2; Mm00477631_m1), a regulator protein involved in tumor progression and resistance to cancer treatments; (*iii*) vascular endothelial growth factor (VEGF-A; Mm00437306_m1), a potent growth factor promoting both angiogenesis and vascular permeability; (*iv*) wingless-type mouse mammary tumor virus (MMTV) integration site family, member 11 (WNT-11; Mm00437327_g1), a signaling protein involved in cancer invasion; and (*v*) ecto-5-nucleotidase (CD73; Mm00501915_m1), an immunoinhibitory protein that plays a major role in tumor growth and metastasis. Total ribonucleic acid (RNA) was extracted from cells using a PureLink™ RNA Mini Kit according to the manufacturer's protocol (Life Technologies). For MCTS cultured in RGD-free cryogels, a bead beater was used to homogenize the samples before RNA extraction. Next, a High-Capacity complementay Deoxyribonucleic Acid (cDNA) Reverse Transcription Kit on a MyCycler (Bio-Rad, Hampton, NH, USA) was used to retrotranscribe mRNA into cDNA according to the manufacturer's recommendation. Finally, the gene expression was assessed using TaqMan Gene Expression Assays (Thermofisher Scientific) on an Mx3005 ​P qPCR System (Agilent, Santa Clara, CA). Relative expression levels were normalized based on hypoxanthine phosphoribosyl transferase (HPRT) expression (housekeeping gene; Mm03024075_m1).

### 2.16 Statistical analysis

All values were expressed as mean ​± ​standard deviation (SD). Statistical analyses were performed using GraphPad Prism Software (La Jolla, CA, USA). Significant differences between groups were analyzed by one-way analysis of variance (ANOVA). Differences were considered significant at ∗p ​< ​0.05 and ∗∗p ​< ​0.01.

## 3 Results

### 3.1 Characterization of RGD-free and RGD-containing HAGM cryogels

**Fig. 2:**
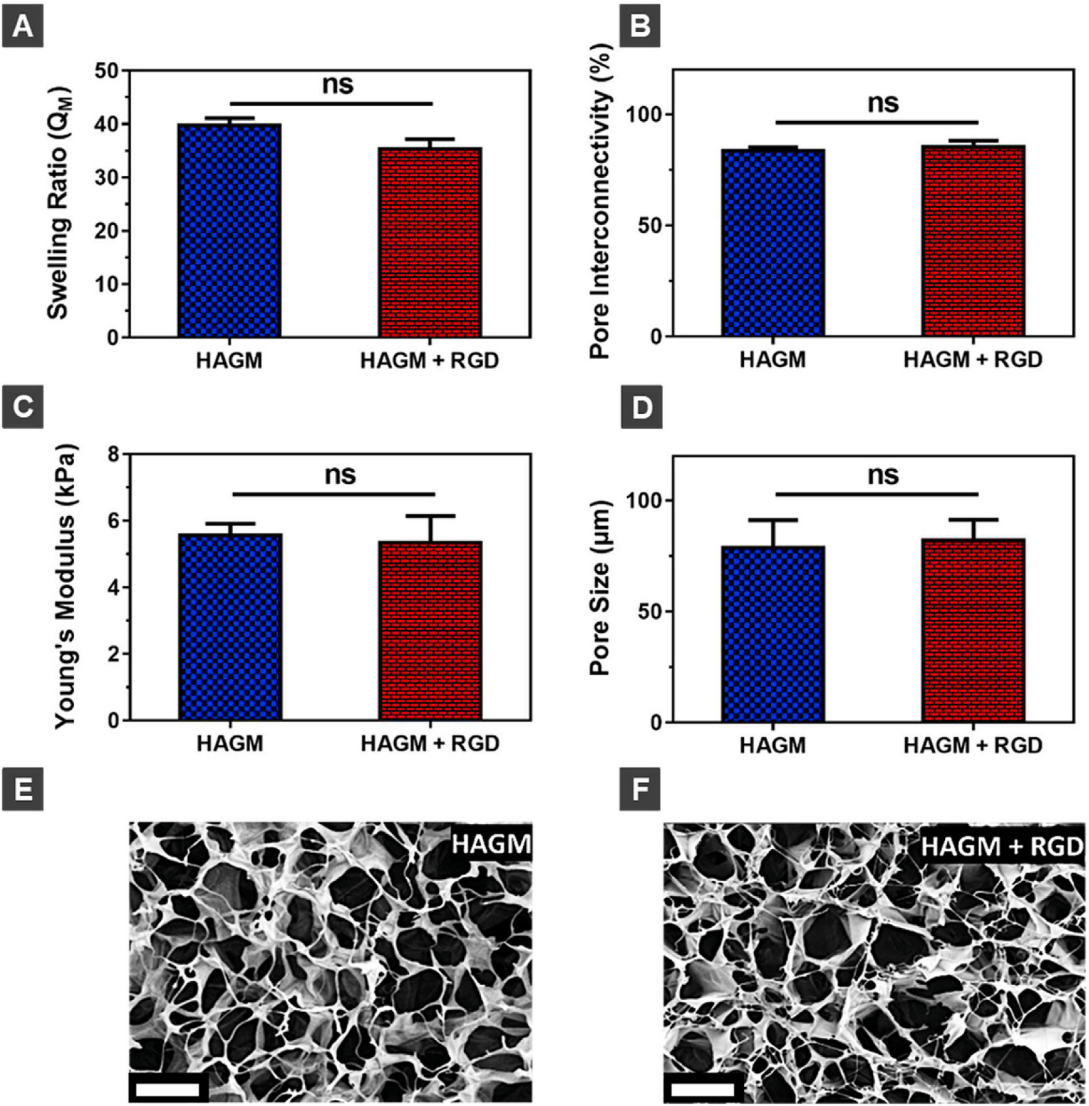
**Characterization of the physical properties of HAGM cryogels.** (A) Swelling ratios, (B) pore connectivity, (C) Young's modulus, and pore size (D) measurements of RGD-free (HAGM) and RGD-containing HAGM (HAGM ​+ ​RGD) cryogels. SEM images of (E) RGD-free and (F) RGD-containing HAGM cryogels showing interconnected macropores. All cryogels were fabricated at −20 ​°C. Values represent the mean ​± ​SD and data were analyzed using one-way ANOVA (n ​= ​5). ns: not significant (p ​> ​0.05). Scale bar ​= ​100 ​μm.

Cryogels were fabricated from HAGM to generate 3D scaffolds with an interconnected macroporous structure that ressembles the native ECM. Additionally, RGD, a synthetic integrin-binding peptide was incorporated into cryogels (RGD-containing cryogels) to promote cell binding. RGD is a widely applied cell-adhesive peptide in tissue engineering because it effectively promotes cell attachment when incorporated into a variety of biomaterials [63]. We compared the structural and physical properties of HAGM cryogels with and without RGD functionalization (Fig. 2). The copolymerization of functionalized RGD with HAGM had no effect on the physical properties (swelling ratio, pore connectivity, Young's modulus, and pore size) of cryogels (Fig. 2A–D). Both types of cryogels (RGD-containing and RGD-free) exhibited a high swelling ratio (∼40) with large (∼75 ​μm) and highly interconnected (>80%) pores. Scanning electron microscope (SEM) confirmed that RGD-free and RGD-containing HAGM cryogels had similar pore sizes and pore size distributions (Fig. 2E and F). Thus, the incorporation of RGD did not have a notable impact on the resulting physical properties of cryogels.

### 3.2 Interactions of individual breast cancer cells with HA-based scaffolds

To investigate the interactions of breast cancer cells with different types of scaffolds, 4T1 cells or 3T3 fibroblasts (control) were cultured within RGD-free HAGM, RGD-containing HAGM, RGD-free PEGDM, and RGD-containing PEGDM cryogels. Cell viability was determined on day 1 and 3 (Figs. S1A–B). Both 4T1 and 3T3 cells exhibited high cell viability within RGD-containing HAGM and RGD-containing PEGDM cryogels. However, within RGD-free HAGM cryogels, high cell viability was only observed for 4T1 cells, whereas the viability was low for 3T3 cells. Furthermore, 4T1 and 3T3 cells showed low viability when cultured within RGD-free PEGDM cryogels. These results suggested that unique and specific interactions occurred between 4T1 cells and HAGM cryogels. To understand the differences between 3T3 and 4T1 cell viability when cultured within RGD-free HAGM cryogels, CD44 surface expression levels were examined by flow cytometry (Figs. S1C–D). In contrast to 3T3 cells, 4T1 cells express a high level of CD44, indicating that the increased viability of 4T1 cells could be associated with CD44-mediated binding to the HA-based cryogel backbone.

**Fig. 3:**
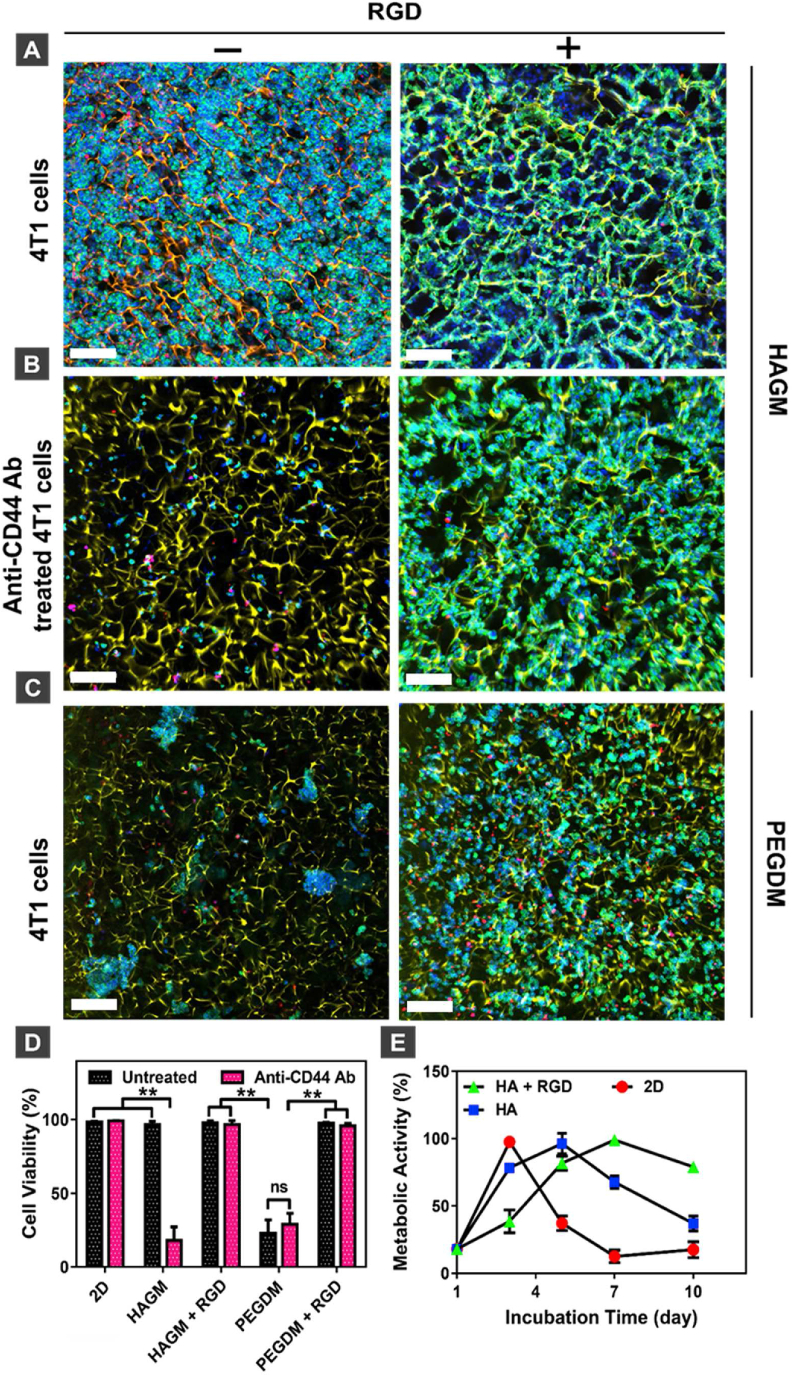
**Culture of individual breast tumor cells on HAGM cryogels.** (A) Confocal microscopy images of 4T1 cells cultured within RGD-free and RGD-containing HAGM cryogels for three days. (B) Confocal microscopy images of anti-CD44-antibody-treated 4T1 cells cultured within RGD-free and RGD-containing HAGM cryogels. (C) Confocal microscopy images of 4T1 cells cultured within RGD-free and RGD-containing PEGDM cryogels. Blue ​= ​nuclei stained with DAPI, red ​= ​dead cells stained with ViaQuant Far Red, green ​= ​actin cytoskeleton stained with Alexa Fluor 488 phalloidin, yellow ​= ​polymer walls stained with rhodamine. (D) In vitro quantitative viability of untreated and anti-CD44-antibody-treated 4T1 cells cultured in 2D and within RGD-free HAGM, RGD-containing HAGM, PEGDM, and RGD-containing PEGDM cryogels for 3 days. Viability of untreated 4T1 cells cultured in 2D was considered the maximum. (E) Metabolic activity of 4T1 cells cultured in 2D and in 3D, either within RGD-free or RGD-containing HAGM cryogels. Maximum absorbance measured for each sample was set to 100% metabolic activity. Values represent the mean ​± ​SD and data were analyzed using one-way ANOVA (n ​= ​5). ∗p ​< ​0.05 and ∗∗p ​< ​0.01. Scale bar ​= ​100 ​μm for (A), 200 ​μm for (B) and (C). (For interpretation of the references to color in this figure legend, the reader is referred to the Web version of this article).

Confocal microscopy was used to image the interactions between 4T1 cells and HAGM cryogels (Fig. 3A). Confocal images showed that 4T1 cells not only interact with RGD-free HAGM cryogels but also uniquely interact with each other, filling the pores of RGD-free HAGM cryogels. Contrastingly, 4T1 cells cultured within RGD-containing HAGM cryogels only interacted with the scaffold and followed the network pattern, leading to a monolayer of cells covering the polymer walls. To confirm CD44-mediated 4T1-HAGM interactions, 4T1 cells were first incubated with anti-CD44 antibodies and then cultured within RGD-free or RGD-containing HAGM cryogels. CD44-blocked 4T1 cells showed no interactions with RGD-free HAGM cryogels (Fig. 3B). Similarly to untreated 4T1 cells, CD44-blocked 4T1 cells interacted with the polymer walls of RGD-functionalized HAGM cryogels. Furthermore, 4T1 cells showed no interactions with RGD-free PEGDM cryogels. In contrast, they bound to the polymer walls of RGD-containing PEGDM cryogels, confirming that 4T1 cells interact specifically with the HA-based cryogel backbone (Fig. 3C). Similarly, 4T1 cells had an aggregated and rounded morphology when cultured in 2D on uncoated cell culture coverslips or mesoporous HAGM hydrogels, but they grew as elongated and stretched single cells on RGD-containing HAGM hydrogels (Fig. S2). For further validation of this phenomenon, a similar study was performed using human MDA-MB-231 breast cancer cells (Figs. S3A–B). As expected, this aggressive triple-negative breast cancer cell line displayed a similar behavior to 4T1 cells. In RGD-free HAGM cryogels, cells formed aggregates and filled the large pores, while they displayed a monolayer of cells coating the pores in RGD-containing HAGM cryogels (Fig. S3A). In both scaffolds, cells showed high viability (Fig. S3B). Moreover, to evaluate cell-cell interactions via the formation of tight junctions, we stained MDA-MB-231 ​cells for the peripheral membrane phosphoprotein ZO-1 (Fig. S3C). Strikingly, we observed high levels of ZO-1 in MDA-MB-231 ​cells cultured in RGD-free HAGM cryogels. However, ZO-1 expression was notably lower in RGD-containing HAGM cryogels suggesting that the peptide may have altered cell-cell interactions.

Because cell-matrix interactions can affect the viability of cells when cultured in cryogels, potential side effects of CD44 blockade on the viability of 4T1 cells in 2D and 3D within cryogel scaffolds were investigated (Fig. 3D). The viability of untreated 4T1 cells in 2D was used as the reference standard (i.e., maximum cell viability). Untreated 4T1 cell viability within RGD-free, RGD-containing HAGM, and RGD-containing PEGDM cryogels was similar to untreated cells grown in 2D. However, untreated 4T1 cells had low viability when cultured within RGD-free PEGDM cryogels. Blocking CD44 receptors significantly reduced cell viability within RGD-free HAGM cryogels, whereas viability was similar to untreated cells across the other conditions. These findings confirmed that the CD44-mediated interactions of 4T1 cells with the HA-based backbone promote cell viability in RGD-free cryogels.

Next, we evaluated the metabolic activity of 4T1 and MDA-MB-231 ​cells when cultured within RGD-free or RGD-containing HAGM cryogels and those cultured in 2D (Figs. 3E and S3D). The temporal pattern of metabolic activity differed among these culture conditions. As previously reported [64], 4T1 and MDA-MB-231 ​cells in 2D exhibited a high metabolic activity that peaked at day 3. The metabolic activity of 4T1 within RGD-free HAGM cryogels was initially higher than those cultured within RGD-containing HAGM cryogels (Fig. 3E). However, the metabolic activity of those cultured in RGD-containing HAGM cryogels peaked at day 7 and remained high up to day 10. Because high metabolic activity can be an indication of a high proliferation rate, these results suggested that RGD initially limited 4T1 cell proliferation within HAGM scaffolds. Interestingly, no differences in the metabolic activity of MDA-MB-231 ​cells were observed between RGD-free and RGD-containing cryogels for up to 10 days (Fig. S3D). Collectively, these results suggest that RGD may compete and alter CD44-mediated interactions with HA and cell-cell signaling, leading to the formation of a monolayer of cells along the polymer walls. In contrast, RGD-free HAGM cryogels promote the spatial organization of CD44-positive breast cancer cells (4T1 and MDA-MB-231) and eventually facilitate tumor microtissue formation.

### 3.3 Optimizing the generation of 4T1 breast MCTS

**Fig. 4:**
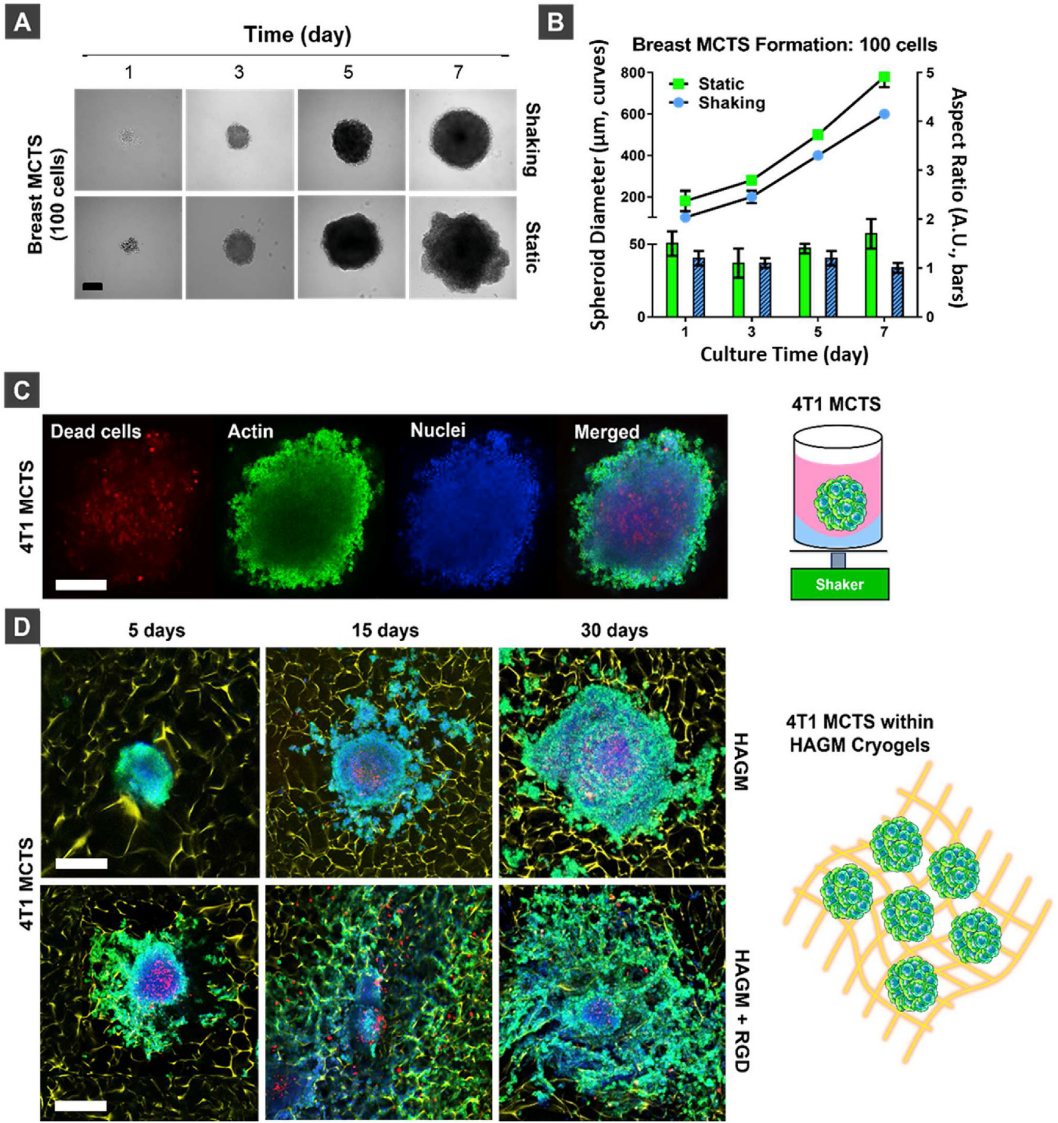
**Optimizing the generation of MCTS and their integration in cryogels.** (A) Brightfield images of breast cancer MCTS cultured for 1, 3, 5, and 7 days in shaking or static conditions starting from 100 ​cells per well. Scale bar ​= ​200 ​μm. (B) Diameter and aspect ratio of MCTS formed starting from 100 ​cells under static or dynamic (orbital shaking) culture conditions after 1, 3, 5 and 7 days. Values represent the mean ​± ​SD (n ​= ​60). Diameter is shown in the line graph (left y-axis) and aspect ratio in the bar graph (right y-axis). (C) Confocal microscopy images of 400 ​μm 4T1 MCTS formed by culturing for 5 days with orbital shaking and starting from 100 ​cells/well, scale bar ​= ​200 ​μm. (D) Confocal microscope images of 400 ​μm 4T1 MCTS formed with orbital shaking for 5 days and then cultured within RGD-free and RGD-containing HAGM cryogels for 5, 15, and 30 days, scale bar ​= ​400 ​μm. Blue ​= ​nuclei stained with DAPI, red ​= ​dead cells stained with ViaQuant Far Red, green ​= ​actin cytoskeleton stained with Alexa Fluor 488 phalloidin, yellow ​= ​polymer walls stained with rhodamine. (For interpretation of the references to color in this figure legend, the reader is referred to the Web version of this article).

After establishing how individual breast cancer cells interact with HAGM cryogels, we engineered a cryogel-supported MCTS platform that recapitulated aspects of breast tumor features in an *in vitro* 3D model [13,57,59]. To obtain standardized and high-quality spheroids, we developed a new method for MCTS generation from low cell numbers. Starting with 100 ​cells/well, we cultured 4T1 cells with constant orbital shaking for 7 days. Compared to 100 ​cells grown under static conditions, 4T1 cells grown with constant shaking formed uniformly shaped MCTS (Fig. 4A). The diameter of 4T1 MCTS increased under both culture conditions, but those formed under orbital shaking remained spherical with aspect ratios of ∼1 throughout the duration of our study (Fig. 4B). After 5 days in culture, the MCTS formed under static conditions were not spherical because some cells in the proliferative layer around the spheroids grew out of the core. This resulted in larger sizes and higher aspect ratios for MCTS formed under static conditions than those formed under orbital shaking (Fig. 4B). Furthermore, confocal microscopy imaging showed that the dense spherical MCTS grown under orbital shaking for 5 days exhibited a necrotic core (Fig. 4C).

Next, we examined whether the initial cell number would affect MCTS morphology under both culture conditions (Figs. S4A–B). Microscopy imaging showed that the size of MCTS increased proportionally to the initial cell number under both static and orbital shaking conditions (Figs. S4A–B). Similarly, the aspect ratios of the MCTS formed under static conditions increased as a function of the initial cell number (Fig. S4B). Interestingly, MCTS formed under orbital shaking grew up to 700 ​μm in size but remained spherical with a dense morphology for up to 7 days.

Further, we evaluated the metabolic activity and physical stability of the MCTS at various time points and their morphology after 10 days in culture. First, MCTS were formed using 100 4T1 cells/well under static or orbital shaking conditions for 3, 5, 7, and 10 days and their metabolic activity was measured using the alamarBlue® assay (Fig. S4C). 4T1 cells cultured in 2D were used as a control. As expected, we did not observe any difference in the metabolic activity across all conditions, indicating a comparable proliferation rate. Then, we evaluated the robustness of 4T1 MCTS formed from 100 ​cells/well at day 5 after micropipette manipulation using brightfield microscopy (Fig. S4D). Unlike the static conditions, MCTS formed under orbital shaking were resistant to cell dissociation when physically manipulated. We also evaluated MCTS morphology after 10 days of culture under orbital shaking, starting from 100 up to 1000 ​cells. By this time, the MCTS lost their spherical shape as cells grew out from the central core, irrespective of the initial cell number (Fig. S5).

Lastly, we compared the cell viability of 4T1 MCTS grown under various combinations of static and dynamic cell culture conditions and then treated with DOX, an anthracycline widely used in breast cancer therapy (Fig. S6A). MCTS formed under orbital shaking and then exposed to Dox under static conditions were the most resistant to the drug, showing a half-maximal inhibitory concentration (IC_50_) of 7.2 ​μM, approximately two-fold higher than IC_50_ for MCTS formed and exposed to the drug under static conditions (Fig. S6B). MCTS exposed to the drug under orbital shaking reduced cell viability both for MCTS formed under static or orbital shaking conditions, most likely due to shaking-mediated penetration of Dox into the cell mass. Taken together, these results suggest that MCTS formed under dynamic conditions are denser than those cultured under static conditions and exhibit an increased drug resistance.

### 3.4 Characterization of 4T1 MCTS-laden HAGM cryogels

Next, we evaluated if MCTS-laden cryogels could reproduce the native breast TME better than those purely grown on agarose-coated wells. MCTS formed under orbital shaking from 100 4T1 cells for 5 days were loaded on RGD-free or RGD-containing HAGM cryogels, and the cell-cell and cell-matrix interactions were imaged using confocal microscopy after 5, 15, and 30 days of culture. The morphology of MCTS and the nature of their interactions with HAGM scaffolds were different depending on the presence or absence of RGD (Figs. 4D and S7). For RGD-free HAGM cryogels, MCTS retained their spherical shape and appeared to adhere to the scaffolds after 5 days of culture. In addition, RGD-free cryogels promoted cell-cell interactions leading to large spheroids displaying a necrotic core after 30 days. On the contrary, MCTS grown on RGD-containing HAGM cryogels lost their spherical shape after only 5 days of culture. 4T1 cells at the outer, proliferative rim of the spheroids established an interaction with the polymer walls and started coating the scaffolds via integrin-mediated cell adhesion and migration. After 30 days of culture, these cells proliferated, forming a monolayer of cells along the polymer network. These distinct cellular behaviors in the proliferative layer of MCTS grown on RGD-free or RGD-containing HAGM cryogels resembled those observed for individual 4T1 cells cultured within the same types of cryogels (Fig. 3A). Collectively, these results suggest that RGD-free cryogels promote the growth of tumor microtissues and appear to be a more suitable platform to model large tumor masses, including those with a more aggressive phenotype.

### 3.5 MCTS-laden HAGM cryogels as a platform for anti-cancer drug screening and genomic analysis

**Fig. 5:**
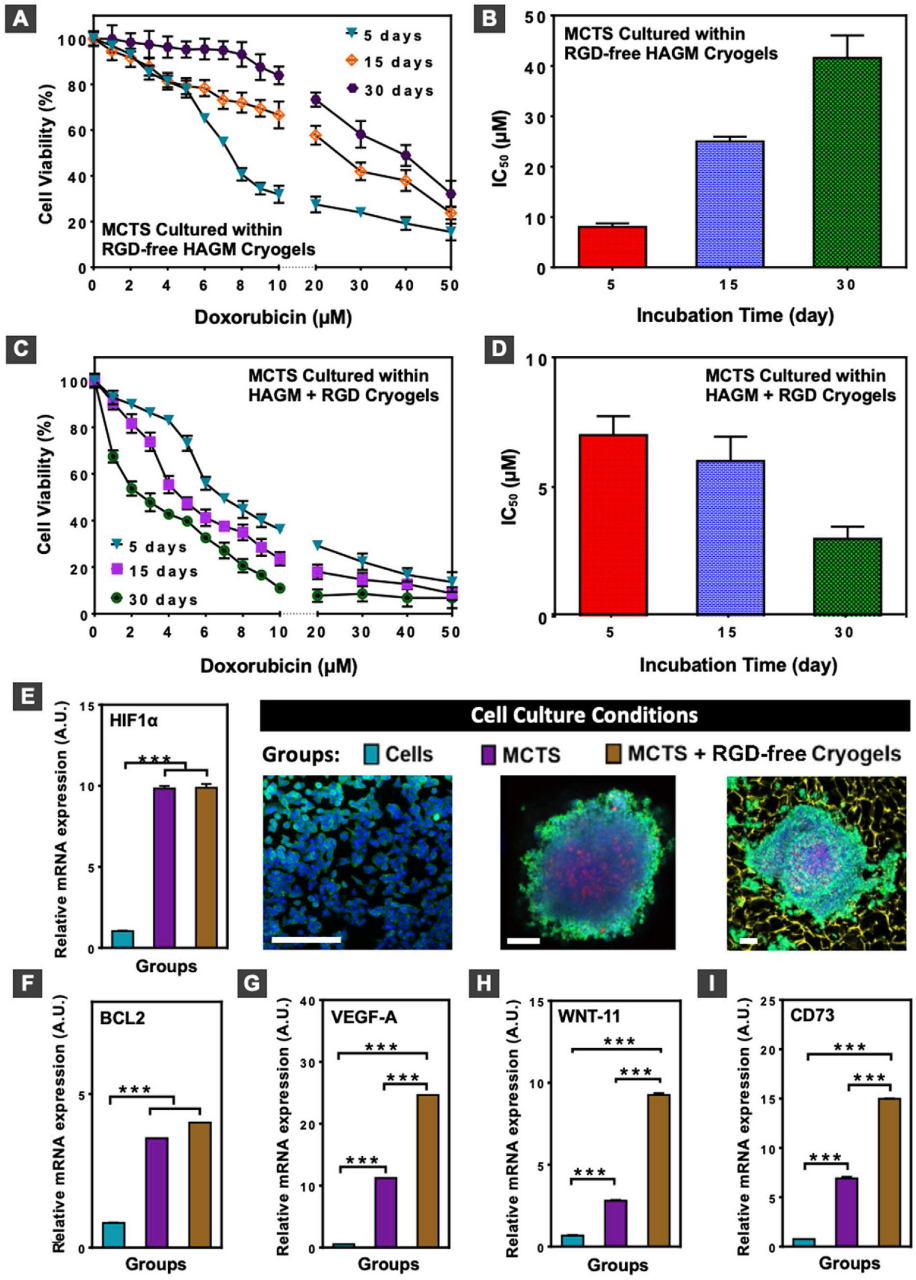
**MCTS cultured in cryogels adopt an aggressive breast tumor cell phenotype and exhibit chemotherapeutic resistance.** (A–D) Cell viability (A, C) and IC_50_ (B, D) of 4T1 MCTS cultured within RGD-free (A, B) or RGD-containing HAGM (C, D) cryogels after 5, 15, and 30 days and then incubated with different concentrations of Dox for 3 days. (E–I) Gene expression encoding HIF1α (E), BCL2 (F), VEGF-A (G) WNT-11 (H), and CD73 (I) of individual 4T1 cells cultured in 2D for 2 days, 4T1 MCTS grown on agarose for 5 days, and 4T1 MCTS cultured within RGD-free HAGM cryogels for 30 days (see corresponding confocal images next to panel E). Transcript abundance was determined by RT-qPCR. Values represent the mean ​± ​SD and data were analyzed using one-way ANOVA (n ​= ​5). ∗p ​< ​0.05 and ∗∗p ​< ​0.01. Scale bar ​= ​200 ​μm.

First, we evaluated the diffusion of solutes within cryogels to ensure that all cancer cells were exposed to the chemotherapeutic agent. Unlike mesoporous HAGM hydrogels, HAGM cryogels allowed rapid and unhindered diffusion of solutes throughout the construct (Fig. S8). Next, we assessed the sensitivity of 4T1 MCTS to Dox when cultured within RGD-free or RGD-containing HAGM cryogels for up to 30 days. We observed substantial cryogel-dependent differences in the sensitivity of 4T1 MCTS to the drug. As expected, MCTS cultured in RGD-free HAGM cryogels acquired a high level of Dox resistance with an IC_50_ of 8 ​μM after 5 days (Fig. 5A–B) that increased over time up to 25 ​μM after 15 days, and 41 ​μM after 30 days. In contrast, MCTS cultured in RGD-containing HAGM cryogels became more susceptible to Dox over longer incubation periods (Fig. 5C), exhibiting an IC_50_ of 7 ​μM after 5 days, 6 ​μM after 15 days, and 3 ​μM after 30 days (Fig. 5D). As the cells in the proliferative layer of 4T1 MCTS grew along the polymer walls further away from the dense tumor masses, they were more likely to be exposed to Dox and eventually undergo apoptosis.

To evaluate the mechanism behind the 4T1 MCTS acquired Dox resistance when cultured in HAGM cryogels, we evaluated the expression of genes associated with cancer aggressiveness and drug resistance. As controls, we analyzed the gene expression of 4T1 cells cultured in 2D or MCTS cultured in agarose coated plates. Unlike 4T1 cells cultured in 2D, both scaffold-free MCTS and MCTS-laden HAGM cryogels displayed increased expression of the gene encoding hypoxia-inducible factor 1α (HIF1α), a key regulator of genes involved in the cellular response to hypoxia (Fig. 5E), and the gene encoding the apoptosis regulator B-cell lymphoma-2 (BCL2) (Fig. 5F). Furthermore, culturing MCTS within RGD-free HAGM cryogels resulted in the highest expression of the genes encoding for VEGF-A (Fig. 5G), WNT-11 (Fig. 5H), and CD73 (Fig. 5I), involved in angiogenesis, cancer invasion, and tumor growth/metastasis, respectively. Collectively, these findings showed that this 3D system represents a valuable step toward emulating an aggressive solid TME that is usually insensitive to most anti-cancer therapies. Culturing MCTS in RGD-free scaffolds enabled the formation of larger and chemotherapy-resistant tumor microtissues.

## 4 Discussion

In vitro tumor models are essential tools in cancer research, enabling the development of new cancer therapies, drug screening platforms, and providing insight into the molecular mechanisms of tumor development and invasion [31,65,66]. In particular, *in vitro* models allow researchers to recapitulate different aspects of the TME, using specific cell types, ECM components, and soluble factors [31,67,68]. Despite advances in therapeutic strategies, anti-cancer drugs often fail in part because preclinical studies lack effective models that incorporate the complexities of the native TME [3,9,69,70]. Conventional 2D assays have been extensively used to assess the role of chemoattractants on cancer cell migration and screen anti-cancer drugs [68,69]. Many of these 2D models do not recapitulate the spatial organization of tumor cells and ECM components found in the native TME [70]. As a result, interest has grown in developing 3D tumor models as promising tools to better understand tumor biology and improve drug screening. In particular, MCTS culture is a popular technique for 3D cancer modeling. As an intermediate between 2D culture and *in vivo* models, MCTS resemble native tumors because they consist of a necrotic core containing dead or quiescent cancer cells, surrounded by a layer of proliferating cells. Furthermore, they recreate oxygen, pH, and nutrient gradients of poorly or non-vascularized tumor tissues [[71], [72], [73], [74]]. Various methods have been investigated to generate MCTS, such as the hanging drop cultures, liquid-overlay techniques, and non-adherent plate cultures [13,14]. However, these methods are often laborious, expensive, or not scalable [71]. Therefore, we developed a simple, cheap, and high throughput method for generating uniform MCTS from breast cancer cells with tunable size. Orbital shaking of 4T1 cells on concave agarose gels resulted in spherical, dense, and resilient MCTS that exhibited high drug resistance, which is a major obstacle to the successful treatment of patients with breast cancer.

To improve 3D models, individual cancer cells and tumor spheroids have been embedded in hydrogels to mimic the ECM of native tissues [75]. However, these gels usually show insufficient porosity for long-term cell survival and proper tumor ECM deposition. Moreover, the spatial distribution of cells in hydrogels is often not uniform, thus generating inconsistent models [54,55]. Cryogels, macroporous 3D scaffolds with high pore connectivity and advantageous physical properties for tissue formation can overcome these limitations [61,[76], [77], [78], [79], [80]]. HAGM cryogels mimic the native tissue microenvironment by providing a 3D physical support, displaying native-like ECM components (HA and RGD, a peptide motif present in fibronectin), and enabling the diffusion of nutrients, oxygen [81], and cellular waste due to their macroporous nature. This unique microenvironment promotes cell-matrix interactions as well as cell motility and spatial organization [82]. These scaffolds were used to evaluate the role of CD44/HA-mediated signaling via cell-cell and cell-matrix interactions with CD44-positive breast cancer cells. CD44 plays a pivotal role in promoting invasion and metastasis of a variety of tumors, including breast cancer [83]. The interconnected macroporous architecture of HAGM cryogels made it possible to observe simultaneous cell-cell and cell-matrix interactions of CD44-positive 4T1 cells in a RGD-free microenvironment. The unique interactions between 4T1 cells and HA, mediated by CD44, resulted in high cell viability and metabolic activity.

Using tissue engineering principles to design functional hydrogels that can recreate *in vivo* TME holds great promise [54]. Tissue-engineered tumor models have been developed to recapitulate key features of the TME while enabling control of environmental factors and over cell responses. The need to design sophisticated models in cancer biology has led to the generation of microenvironments, where complex cell-cell and cell-ECM interactions can occur in a biomimetic fashion [84]. Breast cancer models have been generated on various biomaterials, including silk, alginate, chitosan, and Matrigel. However, most of these materials are not native of human breast tumor ECM and therefore fail to accurately mimic the TME [85]. Collagen has also been extensively used as a bioactive polymer in 3D tumor models to study breast cancer invasion and metastasis. Although the use of collagen is physiologically relevant, these scaffolds contain RGD ligands [86]. Our study indicated that integrin-mediated tumor cell binding to these ligands may prevent the formation of solid-like tumor masses. Furthermore, the use of collagen and collagen derivatives, such as gelatin, makes it difficult to study the impact of other critical tumor ECM components such as HA, which remains underinvestigated [86]. HA is a naturally occurring glycosaminoglycan known to play a major role in cancer progression, and its accumulation in the TME has been associated with poor clinical outcomes in various malignancies, including breast cancer [[87], [88], [89]]. Our MCTS-laden HAGM cryogel platform could be leveraged as an *in vitro* tool to further investigate the role of HA on promoting epidermal growth factor receptor (EGFR)-mediated signaling pathways as well as breast tumor metastasis and chemoresistance. In addition, HAGM at various molecular weights or degrees of methacrylation could be used to fine tune the mechanical properties of cryogels and subsequently evaluate the effect of substrate stiffness on cancer cell behavior and motility [90].

The molecular profile of tumor cells can accurately predict tumor aggressiveness. Markers for hypoxic and metastatic signaling are associated with poor clinical outcomes and low response rates to anti-cancer drugs [60]. A hypoxic microenvironment, which is critical during cancer development, has been identified to play a key role in promoting molecular processes involved in breast cancer metastasis [89]. HIF proteins regulate the transcription of several genes involved in key steps of the metastatic process such as angiogenesis, ECM modulation, cell migration, and adhesion [91]. High fractions of hypoxic cells and increased expression of the gene encoding HIF1α in scaffold-free and 4T1 MCTS-laden HAGM cryogels compared to those in 4T1 cells cultured in 2D indicated increased cellular hypoxia within MCTS, which may lead to more aggressive, metastatic, and therapeutically resistant tumor phenotypes. Furthermore, overexpression of WNT-11 has been associated with migration and metastasis of breast cancer cells [92]. Additionally, high expression of anti-apoptotic protein BCL2 promotes tumor progression and resistance to cancer treatments [93]. Finally, the growth of large, solid tumor microtissues on RGD-free HAGM cryogels, along with HA-mediated CD44 activation, is probably responsible for the overexpression of genetic markers for hypoxia and tumor aggressiveness, including HIF1α, WNT-11, BCL2, CD73, and VEGF [94,95]. Future *in vitro* studies with RGD-free HAGM cryogel-based tumor models could further elucidate the role of HA-mediated CD44 activation in breast tumor progression and metastasis and could investigate the specific variant of CD44 isoforms involved in CD44/HA-mediated signaling pathways. Additionally, the upregulation of HA-mediated motility receptor (RHAMM) promotes breast cancer progression [96], and our engineered 3D model could be used for investigating the role of RHAMM in CD44-positive breast cancer cell progression and metastasis. Lastly, it has been reported that cancer-associated fibroblasts contribute to therapy resistance and metastasis, and endothelial cells play an active role in promoting breast cancer proliferation [97]. With that in mind, HAGM cryogels could be used for MCTS co-cultures of breast cancer cells, fibroblasts, and endothelial cells to better model breast tumor heterogeneity and complexity [98,99].

## 5 Conclusion

Our study showed that, when cultured in RGD-containing HAGM cryogels, the RGD peptide interfered with the cellular behavior of 4T1, a CD44-positive metastatic breast cancer cell line. In contrast, 4T1 cells interacted with RGD-free HAGM cryogels in a CD44-dependent manner to encourage cell-cell interactions and the spatial organization of 4T1 cells. RGD promoted cell-scaffold interactions over cell interactions and impeded CD44/HA-mediated signaling pathways. Furthermore, dense MCTS were generated with consistent size and shape using a simple method yet high throughput. Loading MCTS on RGD-free HAGM cryogels resulted in the formation of large, highly aggressive, and DOX-resistant tumor microtissues. This novel tumor model has great potential for preclinical drug testing and could be used to further understand the mechanisms of CD44-positive cancer cell progression in an HA-rich TME.

## Credit authorship contribution statement

Mahboobeh Rezaeeyazdi: Conceptualization, Methodology, Validation, Investigation, Data curation, Visualization, Writing – original draft. Thibault Colombani: Methodology, Validation, Investigation, Writing – review & editing. Loek J. Eggermont: Writing – review & editing. Sidi A. Bencherif: Conceptualization, Writing – review & editing, Supervision, Project administration, Funding acquisition.

## Data availability

Complete data from this study can be obtained by contacting the corresponding author upon reasonable request.

## Declaration of competing interest

The authors declare that they have no known competing financial interests or personal relationships that could have appeared to influence the work reported in this paper.
