# Supplementary material for: Engineering hyaluronic acid-based cryogels for CD44-mediated breast tumor reconstruction
Source: Mater Today Bio. 2022 Jan 24;13:100207. doi: 10.1016/j.mtbio.2022.100207 (PMC8844817; doi:10.1016/j.mtbio.2022.100207)
Supplement: Multimedia component 1 [file mmc1.docx]

Supplementary Information

**Engineering Hyaluronic Acid-based Cryogels for CD44-mediated Breast Tumor Reconstruction**

Mahboobeh Rezaeeyazdi^†^, Thibault Colombani^†^, Loek J Eggermont, and Sidi A. Bencherif^*^

Dr. M. Rezaeeyazdi
Department of Chemical Engineering, Northeastern University, Boston, MA, 02115, USA

Dr. T. Colombani

Department of Chemical Engineering, Northeastern University, Boston, MA, 02115, USA

Dr. L. J. Eggermont

Department of Chemical Engineering, Northeastern University, Boston, MA, 02115, USA

Prof. S. A. Bencherif

Department of Chemical Engineering, Northeastern University, Boston, MA, 02115, USA

Department of Bioengineering, Northeastern University, Boston, MA, 02115, USA

Harvard John A. Paulson School of Engineering and Applied Sciences, Harvard University, Cambridge, MA, 02138, USA

^*^E-mail: [s.bencherif@northeastern.edu](mailto:s.bencherif@northeastern.edu)

^†^Authors contributed equally


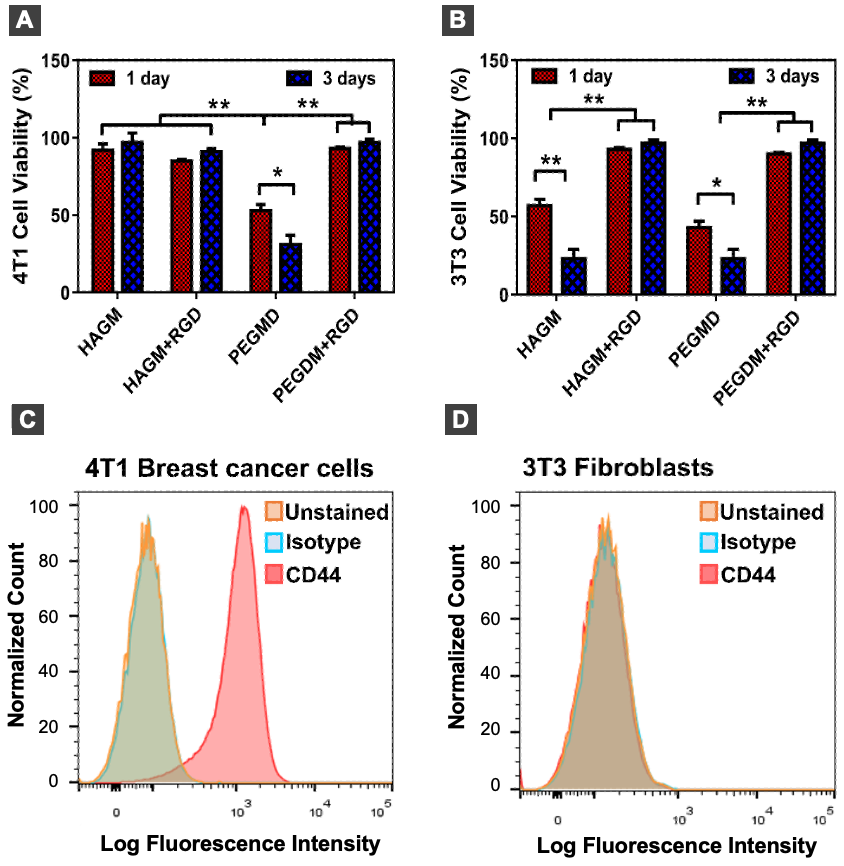


**Figure S1. CD44-mediated interaction of 4T1 breast cancer cells with HAGM cryogels.** Viability of (A) 4T1 cells and (B) 3T3 fibroblasts cultured within RGD-free and RGD-containing HAGM, as well as RGD-free and RGD-containing PEGDM cryogels for 1 and 3 days. Flow cytometry histograms of (C) 4T1 cells and (D) 3T3 fibroblasts stained with brilliant violet 605-anti-mouse/human CD44 or brilliant violet rat IgG2b isotype antibody. Values represent the mean ± SD and data were analyzed using one-way ANOVA (n = 5). *p < 0.05 and **p < 0.01.


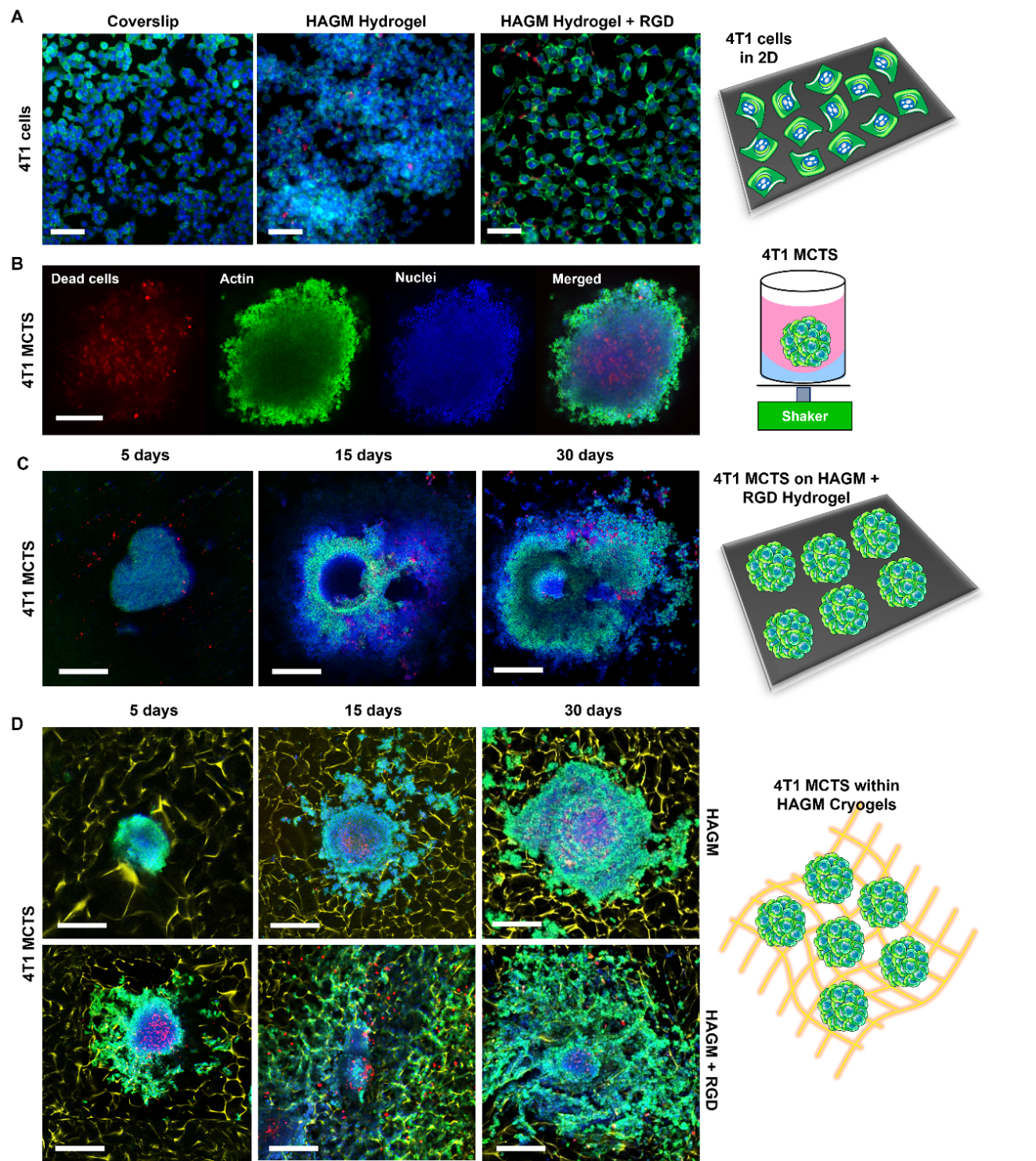


**Figure S2. 4T1 breast cancer cells cultured in 2D.** Fluorescent microscopy images of 4T1 cells on coverslip and RGD-free and RGD-containing HAGM hydrogels for 3 days, scale bar = 50 µm. Blue = nuclei stained with DAPI, red = dead cells stained with ViaQuant Far Red, green = actin cytoskeleton stained with Alexa Fluor 488 phalloidin, yellow = polymer walls stained with rhodamine.

*
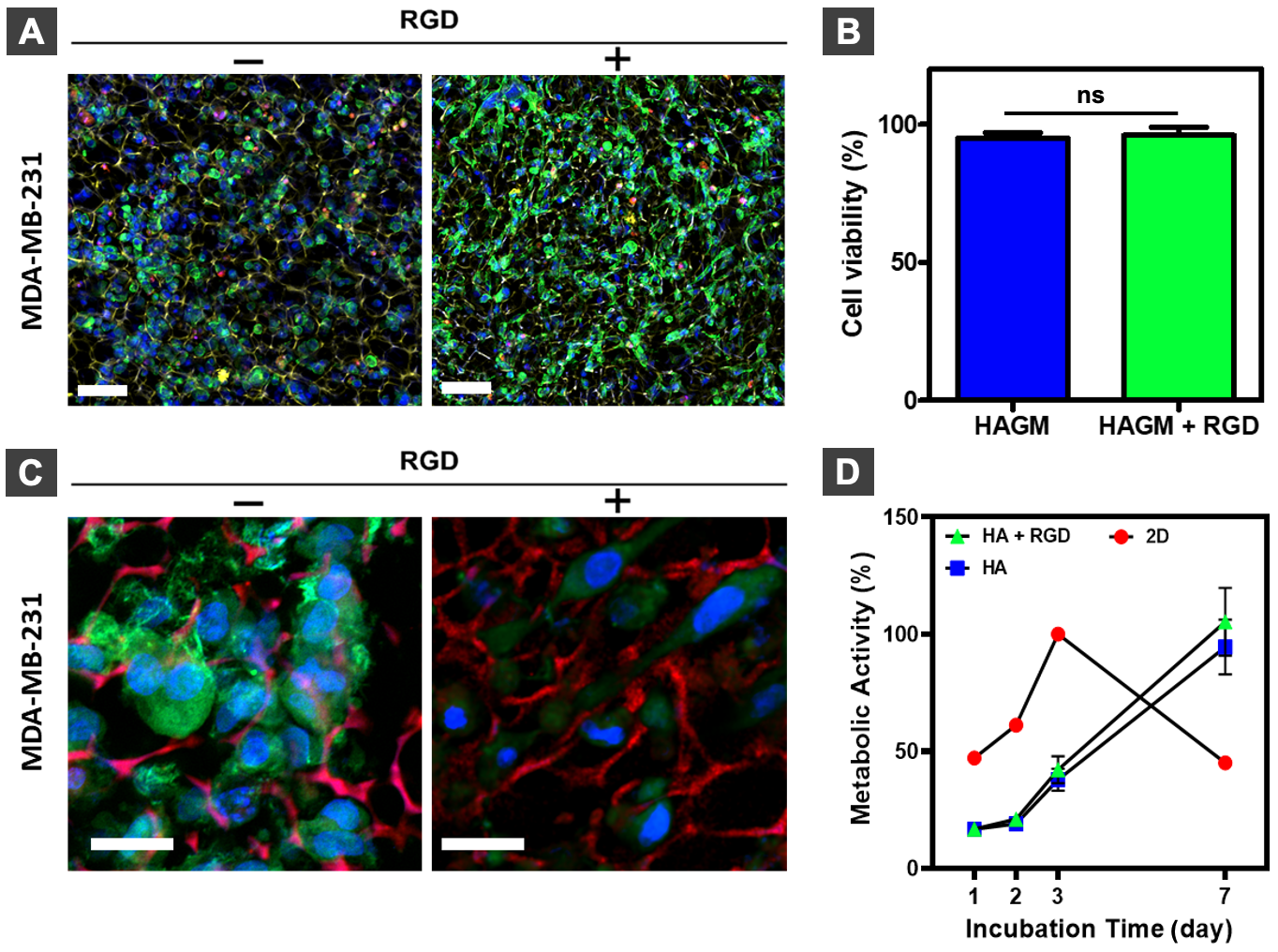
*

**Figure S3.** **Culture of individual human breast tumor cells on HAGM cryogels.** (A) Confocal microscopy images of MDA-MB-231 cells cultured within RGD-free and RGD-containing HAGM cryogels for 3 days. Blue = nuclei stained with DAPI, red = dead cells stained with ViaQuant Far Red, green = actin cytoskeleton stained with Alexa Fluor 488 phalloidin, yellow = polymer walls stained with rhodamine. (B)Viability of MDA-MB-231 cells cultured within RGD-free and RGD-containing HAGM for 3 days. (C) Confocal microscopy images of MDA-MB-231 cell-laden cryogels depicting cell-cell interactions within RGD-free and RGD-containing HAGM cryogels after 3 days of culture. Blue = nuclei stained with DAPI, green = ZO-1 stained with Anti-ZO-1 Alexa Fluor 488, red = polymer walls stained with rhodamine. (D) Metabolic activity of MDA-MB-231 cells cultured in 2D and in 3D, either within RGD-free or RGD-containing HAGM cryogels. Maximum absorbance measured for each sample was set to 100% metabolic activity. Values represent the mean ± SD and data (B) were analyzed using one-way ANOVA (n = 5). ns: not significant (p > 0.05). Scale bar = 100 µm (A) and 25 µm (C).


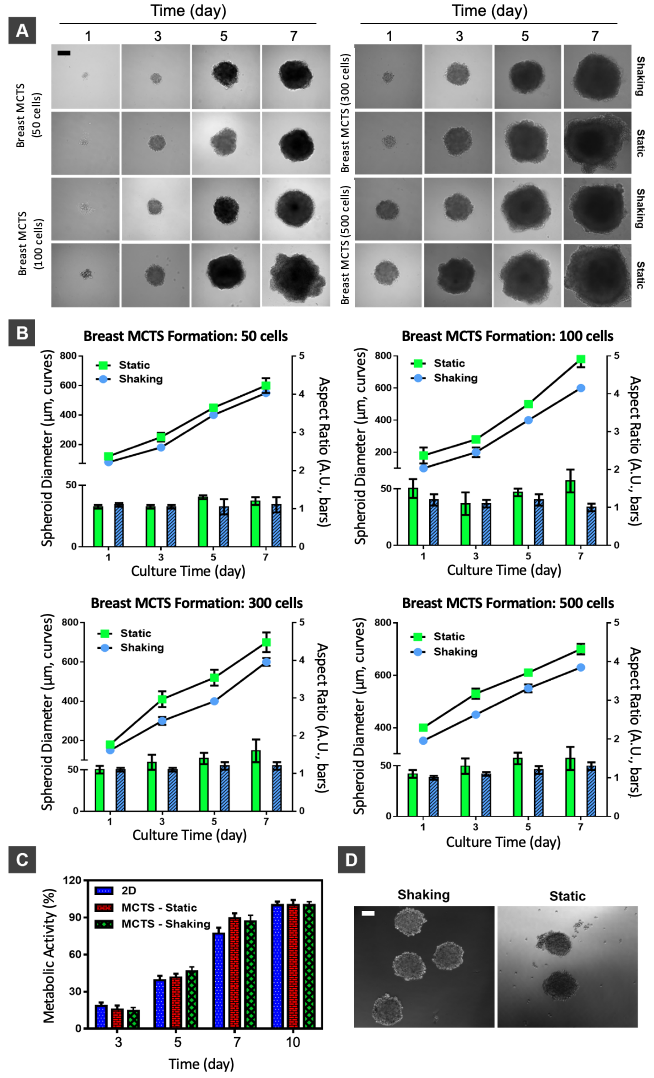


**Figure S4**. **Efficient formation of breast MCTS from low density of cells through orbital shaking.** (A) Brightfield images of breast cancer MCTS cultured for 1, 3, 5, and 7 days under orbital shaking or static conditions starting with 50, 100, 300, and 500 cells per well. (B) Diameter and aspect ratio of MCTS formed starting from 50, 100, 300, and 500 cells cultured for 1, 3, 5, and 7 days under orbital shaking or static conditions. Values represent the mean *±* SD (n = 60). (C) Change in metabolic activity of 4T1 breast cancer cells cultured in 2D and in the form of MCTS grown under static conditions or with orbital shaking. Maximum absorbance measured for each sample was set to 100% viability for that sample*.* (D) Brightfield images of 4T1 breast cancer MCTS transferred by pipette after 5 days of culture starting with 100 cells and grown under static or orbital shaking conditions. Scale bar = 200 µm.


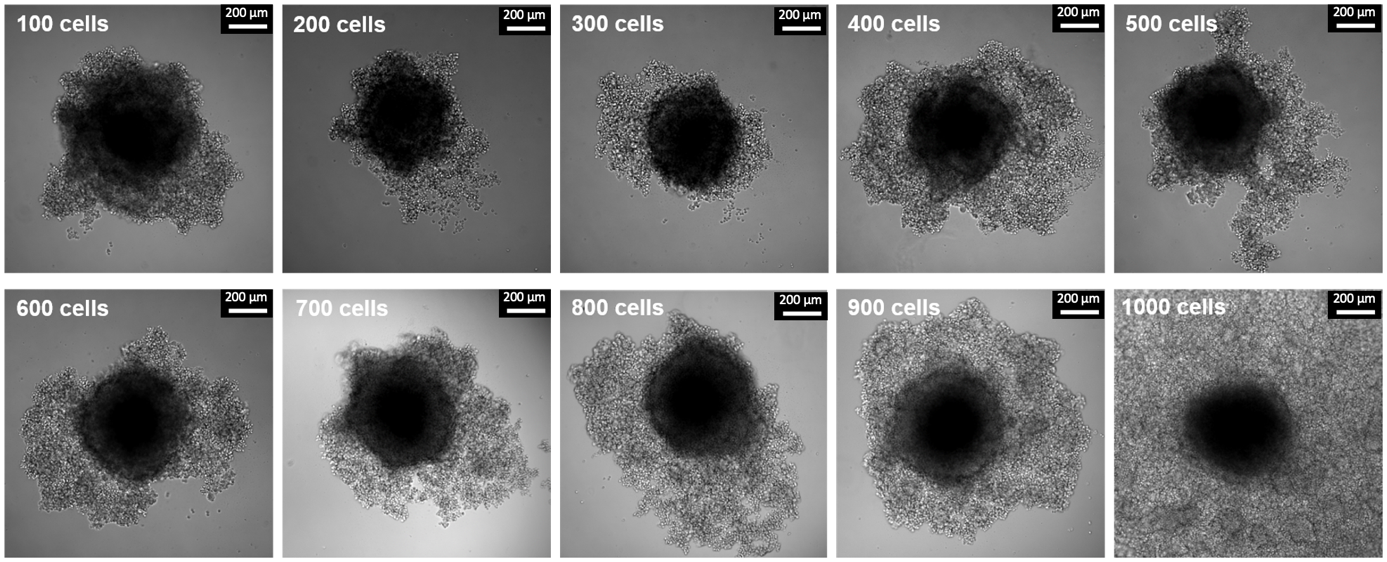


**Figure S5**. **Morphology of breast MCTS formed on agarose gels through orbital shaking.** Brightfield images of breast cancer MCTS cultured for 10 days on agarose gel through orbital shaking. The initial cell number (from 100 up to 1000 cells) is indicated. Scale bar = 200 µm.


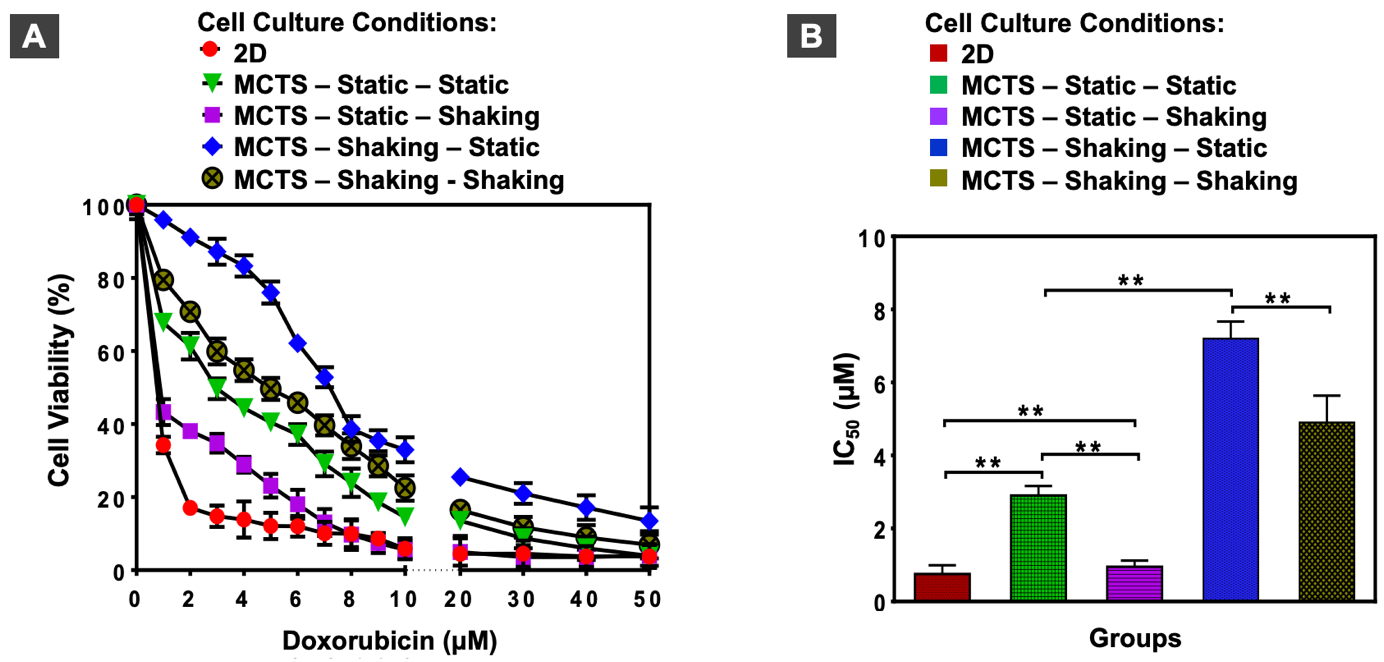


**Figure S6. Breast MCTS as an in vitro anti-cancer drug screening platform.** (A) Viability and (B) IC50 of 4T1 cells in 2D and in MCTS grown from 100 cells under orbital shaking and static conditions, incubated with different concentrations of doxorubicin for 3 days under orbital shaking and static conditions. Values represent the mean ± SD and data were analyzed using one-way ANOVA (n = 5). *p < 0.05 and **p < 0.01.


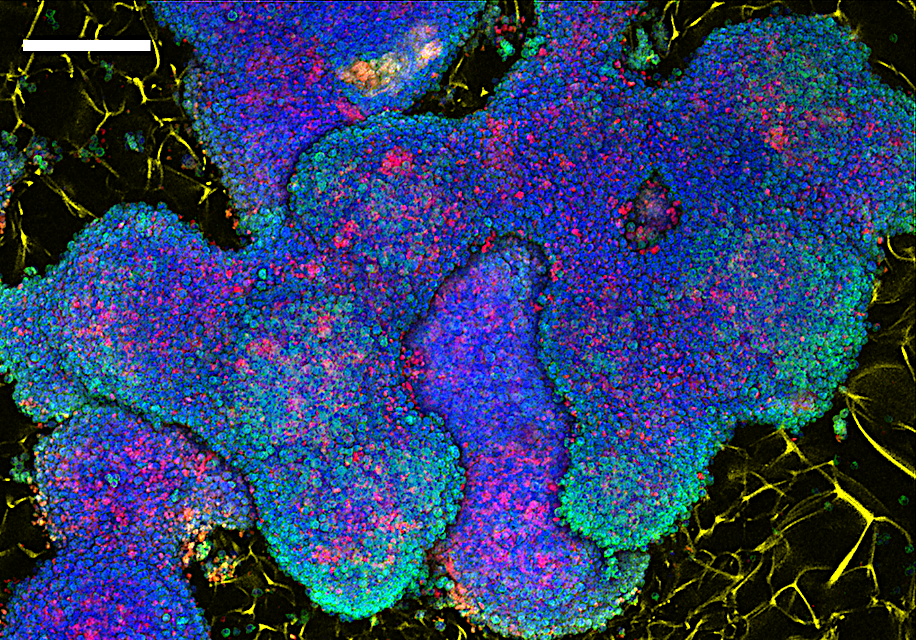


**Figure S7**. **Integration of breast cancer MCTS within cryogels.** Confocal microscope image of 400-µm 4T1 MCTS formed with orbital shaking for 5 days and then cultured within RGD-free HAGM cryogels for 5 days. Blue = nuclei stained with DAPI, red = dead cells stained with ViaQuant Far Red, green = actin cytoskeleton stained with Alexa Fluor 488 phalloidin, yellow = polymer walls stained with rhodamine. Scale bar = 400 µm.


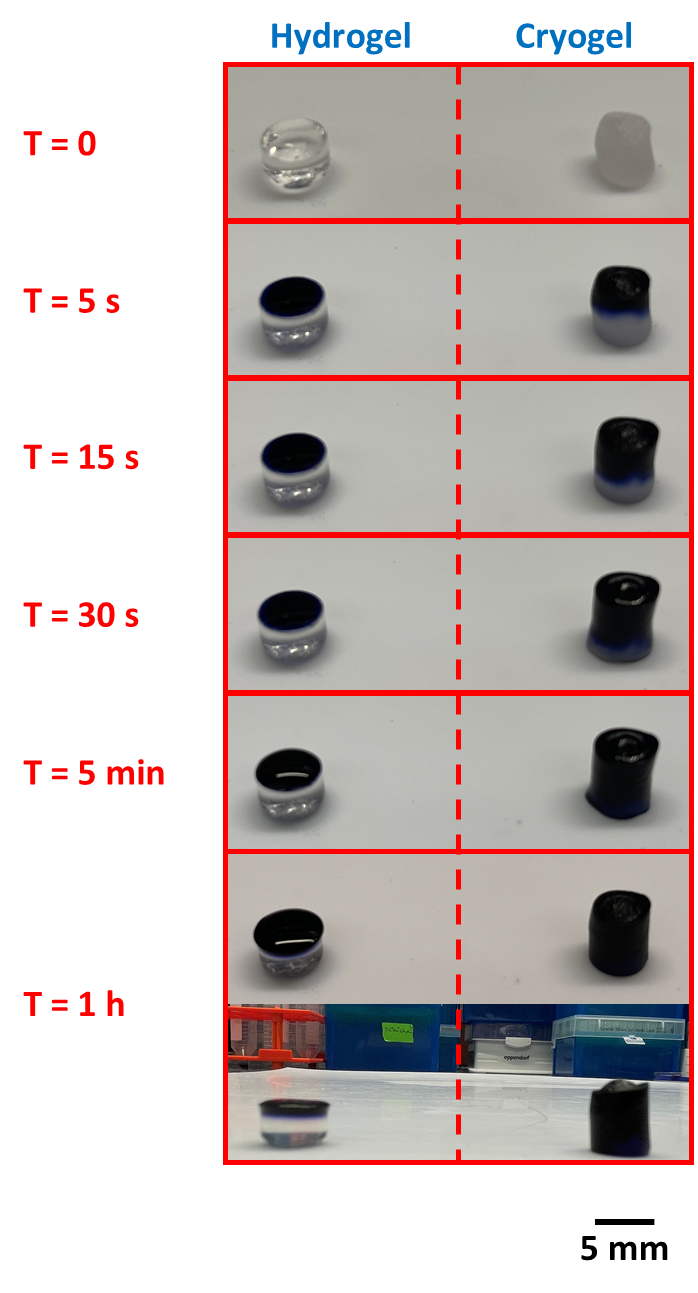


**Figure S8**. **Diffusion of** **Trypan blue dye within hydrogels and cryogels.** Photographs showing the diffusion of Trypan blue (200 µL, dark blue dye) within hydrogels (left column) and cryogels (right column) at various time points (T = 0 s, 5 s, 15 s, 30 s, 5 min, and 1 h). Photos are representative of n = 5 scaffolds.
